# Complete Genome Sequence of *Streptomyces* Phage ϕRKBJ001 Isolated from Prince Edward Island, Canada

**DOI:** 10.1128/mra.01174-21

**Published:** 2022-02-17

**Authors:** Benjamin P. Johnston, Bradley Haltli, Russell G. Kerr

**Affiliations:** a Department of Chemistry, University of Prince Edward Island, Charlottetown, PE, Canada; b Department of Biomedical Sciences, University of Prince Edward Island, Charlottetown, PE, Canada; c Nautilus Biosciences CRODA, Regis and Joan Duffy Research Centre, Charlottetown, PE, Canada; DOE Joint Genome Institute

## Abstract

We reported here the complete genome sequence of *Streptomyces* phage ϕRKBJ001 that was isolated from a saltwater marsh on Prince Edward Island, Canada, using the *Streptomyces* sp. strain RKBHB0173. Based on electron microscopy and genomic analysis, this phage belongs to the *Siphoviridae* family and the BN *Streptomyces* phage cluster.

## ANNOUNCEMENT

*Streptomyces* spp. are a rich source of natural products (NPs), but, in the laboratory setting, the majority of biosynthetic gene clusters (BGCs) that encode these NPs remain dormant (1). As part of an effort to better understand how bacteriophages impact cryptic BGCs expression in *Streptomyces* bacteria, we used *Streptomyces* sp. strain RKBHB0173 (GenBank accession no. MW494619) to isolate phage ϕRKBJ001 from a sediment sample collected from a saltwater marsh (depth, 0.15 m; temperature, 26.3°C; pH, 7.2; salinity, 24.5 ppt) near Kinlock Beach on Prince Edward Island, Canada (46.199302 N, 63.0812886 W) in August of 2019.

PhiRKBJ001 was enriched using RKBHB0173 and isolated by following established protocols (2, 3), with minor adjustments. Briefly, the sediment sample was centrifuged at 1000 × *g* to remove excess water and a slurry of 10 g of sediment in 20 mL of phage buffer (10 mM Tris-HCl [pH 7.5], 10 mM MgSO_4_, 68 mM NaCl) was agitated at 300 RPM for 1 h to extract phage from the sediment. Samples were vortexed for 10 s and clarified by centrifugation followed by filtration (0.22 μm cellulose acetate). Extracted phage was enriched by incubating with RKBHB0173 spores at 30°C and 200 RPM for 2 days in 1:1 ratio with double strength International *Streptomyces* Project-2 (ISP2) growth medium (4) supplemented with 18% Instant Ocean® Sea Salt and 1 mM CaCl_2_.

Pure stocks of ϕRKBJ001 were obtained by 3 rounds of plaque purifications by streak plating and a high-titer stock (5 × 10^12^ PFU/mL) was generated from CsCl density-gradient ultracentrifugation. Genomic DNA was extracted with phenol-chloroform based on published protocols (3) and DNA purity and integrity were assessed spectrophotometrically and by gel electrophoresis. Library preparation and sequencing were conducted by the center d'expertise et de services at Genome Québec using the NEBNext® Ultra II DNA Library Prep kit for Illumina (NEB BioLabs) and the Illumina MiSeq platform to produce a total of 197,206 paired-end 250-bp reads. Geneious Prime software (version 2020.2.2) was used to trim the paired reads with BBDuk (version 38.84) using the following settings: kmer length = 27, minimum quality and overlap = 20, and minimum length = 50. *De novo* genome assembly was performed on 157,246 trimmed reads with the Geneious Assembler module using the default settings and the option to circularize contigs if ends match with 3 or more sequences selected. The 153,548 reads were assigned to one contig that was circularized into a consensus sequence with an average read coverage of 443×. The assembled genome was annotated in Geneious Prime with the Glimmer 3 plugin (version 1.5) and with web software GeneMarkS (version 4.28) (5). The locations of start codons were adjusted based on sequence homology to related phage open reading frames (ORFs) while maintaining an overlap of less than 40 bp with the upstream ORF, if applicable. Functional ORFs were assigned based on results from the nonredundant protein sequences search database in BLASTP (6) and the default/structural domain database in HHpred (7), using default settings. Web software tRNAscan-SE (8) was used to search for the presence of tRNAs.

**FIG 1:**
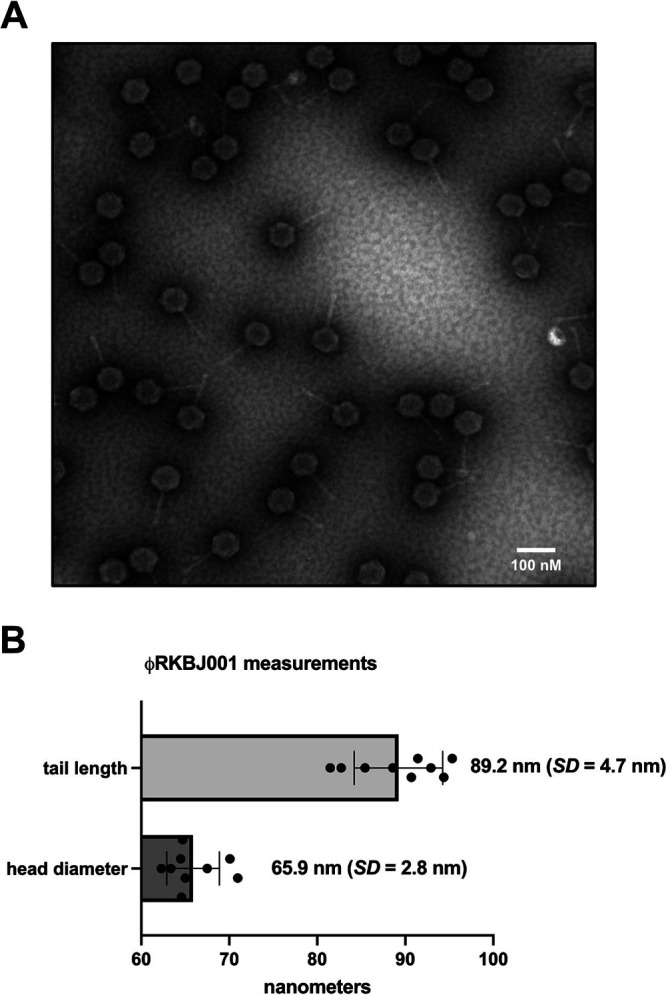
Electron microscopic analysis of ϕRKBJ001. (A) Purified ϕRKBJ001 phage was fixed in 2.5% glutaraldehyde and negatively stained with 2% aqueous uranyl acetate. Samples were imaged on a JEOL 1230 transmission electron microscope at 80 kV with a Hamamatsu ORCA-HR digital camera. The scale bar is 100 nm. (B) The average and standard deviation of tail length and head diameter of nine phages were measured by first determining the digital length of the scale bar in pixels using Fiji software (version 2.3) (9).

PhiRKBJ001 had a circularly permuted DNA genome of 69,373 bp with a GC content of 63.7%. Based on nucleotide sequence homology, ϕRKBJ001 clusters with the lytic *Streptomyces* BN cluster (Table 1) and had the closest sequence similarity to phage Wentworth. Electron micrographs confirmed that ϕRKBJ001 belongs to the *Siphoviridae* family (Fig. 1). The ϕRKBJ001 genome is devoid of any tRNAs, which was consistent with other phages in the BN cluster. Thirty percent of the 109 annotated ORFs were identified as functional phage proteins, while 5 of the hypothetical proteins were unique to ϕRKBJ001. Further studies of ϕRKBJ001 and its associated genes will be performed to determine their impact on the regulation of host BGCs.

**TABLE 1:** Genomic characteristics of ϕRKBJ001 and *Streptomyces* phages in BN cluster

Phage	GenBank accession no.	Genome size (bp)	GC content (%)	No. of ORFs	Coverage^*a*^ with ϕRKBJ001 (%)	Identity^*a*^ with ϕRKBJ001 (%)	Isolation host	Source or reference
ϕRKBJ001	MT936332	69,373	63.7	109	100	100	*Streptomyces*. sp. strain RKBHB0173	This study
Dryad	MT498037	68,994	63.8	109	73	83.71	S. viridochromogenes	GenBank
Gibson	MK305891	69,439	64.4	105	80	82.21	S. griseus	GenBank
Lizz	MZ648036	69,287	64.2	103	85	82.62	S. viridochromogenes	GenBank
PHTowN	MT498053	69,271	64.3	104	80	83.08	S. viridochromogenes	GenBank
Rooney	MZ648033	69,514	64.3	105	82	82.33	S. viridochromogenes	GenBank
ShakeNBake	MT897908	69,299	64.2	104	81	82.74	S. viridochromogenes	GenBank
Wentworth	MH019216	68,260	64.1	103	83	91.82	S. griseus	GenBank
Yara	MH019215	68,671	63.9	105	22	83.77	S. toxytricini	GenBank

a Percent coverage and identity are based on nucleotide alignments obtained from BLAST.

### Data availability.

The annotated genome of ϕRKBJ001 was deposited in GenBank with accession no. MT936332 and the sequencing data have been deposited in the Sequence Read Archive with accession no. SRX9062343 under BioProject accession no. PRJNA660661.

## References

[1] Rutledge PJ, Challis GL. 2015. Discovery of microbial natural products by activation of silent biosynthetic gene clusters. Nat Rev Microbiol 13:509–523. doi:10.1038/nrmicro3496. 26119570

[2] Wommack KE, Williamson KE, Helton RR, Bench SR, Winget DM. 2009. Methods for the isolation of viruses from environmental samples. Methods Mol Biol 501:3–14. doi:10.1007/978-1-60327-164-6_1. 19066805

[3] Russell DA, Hatfull GF. 2017. PhagesDB: the actinobacteriophage database. Bioinformatics 33:784–786. doi:10.1093/bioinformatics/btw711. 28365761 PMC5860397

[4] Shirling EB, Gottlieb D. 1966. Methods for characterization of *Streptomyces* species. Int J Syst Bacteriol 16:313–340. doi:10.1099/00207713-16-3-313.

[5] Besemer J, Lomsadze A, Borodovsky M. 2001. GeneMarkS: a self-training method for prediction of gene starts in microbial genomes. Implications for finding sequence motifs in regulatory regions. Nucleic Acids Res 29:2607–2618. doi:10.1093/nar/29.12.2607. 11410670 PMC55746

[6] Gish W, States DJ. 1993. Identification of protein coding regions by database similarity search. Nat Genet 3:266–272. doi:10.1038/ng0393-266. 8485583

[7] Hildebrand A, Remmert M, Biegert A, Söding J. 2009. Fast and accurate automatic structure prediction with HHpred. Proteins 77:128–132. doi:10.1002/prot.22499. 19626712

[8] Lowe TM, Eddy SR. 1997. tRNAscan-SE: a Program for Improved Detection of Transfer RNA Genes in Genomic Sequence. Nucleic Acids Res 25:955–964. doi:10.1093/nar/25.5.955. 9023104 PMC146525

[9] Schindelin J, Arganda-Carreras I, Frise E, Kaynig V, Longair M, Pietzsch T, Preibisch S, Rueden C, Saalfeld S, Schmid B, Tinevez JY, White DJ, Hartenstein V, Eliceiri K, Tomancak P, Cardona A. 2012. Fiji: an open-source platform for biological-image analysis. Nat Methods 9:676–682. doi:10.1038/nmeth.2019. 22743772 PMC3855844

